# Study on the path of combining music and digital health technology to promote the health of older adult groups

**DOI:** 10.3389/fpubh.2025.1633924

**Published:** 2026-01-26

**Authors:** Chuang Ma, Bo Hu, Shixue Chen, Xiaomei Ma

**Affiliations:** 1 School of Music, Southwest University, Chongqing, China; 2 Guang'anmen Hospital South Campus, China Academy of Chinese Medical Sciences, Beijing, China; 3 Department of Oncology, The First Affiliated Hospital of Chongqing Medical University, Chongqing, China

**Keywords:** deep learning, Health, Health of older adult groups, health score, music and digital health

## Abstract

**Objective:**

As the global population ages, non-pharmacological interventions such as personalized music therapy show promise for wellbeing in older adults. We propose the Fusion-Attentive Temporal Network (FAT-Net). This dual-stream model processes minute level heart-rate and music on/off data alongside daily summary features to predict a composite health score.

**Methods:**

Data from 92 participants over 45 ± 10 days were augmented fourfold using jittering, time-warping, magnitude scaling, and SMOTE. The temporal stream uses Conv1D, BiLSTM, and self-attention pooling. The summary stream uses a three-layer MLP. Cross-modal attention fuses both embeddings.

**Results:**

Over ten runs, FAT-Net achieved RMSE = 0.35 ± 0.005 (22.7% reduction vs. Random Forest), MAE = 0.28 ± 0.005 (19.5% reduction), and *R*^2^ = 0.87 ± 0.008 (17.3% improvement). Pearson's *r* between predictions and true values was 0.93.

**Conclusion:**

FAT-Net's attention-based fusion provides a robust, interpretable approach for forecasting daily wellbeing in older adults.

## 1 Introduction

### 1.1 Background and significance

As the global population ages, the need for scalable, proactive solutions to support health and wellbeing among older adults has become increasingly urgent. By 2050, adults aged 60 and over are projected to comprise 22% of the global population (1). This demographic shift is accompanied by a rise in chronic diseases and cognitive decline (2, 3), placing immense pressure on healthcare systems. In this context, non-pharmacological interventions such as music therapy have shown promise in promoting mental and emotional well-being, particularly by enhancing mood, memory, and social engagement in older adults (1, 3, 4). Additionally, music listening has been linked to modulation of physiological indicators like heart rate variability (HRV) and stress biomarkers (4, 5).

Advancements in wearable technology enable the continuous collection of physiological signals such as heart rate and HRV (6, 7). These data offer new opportunities to develop personalized digital therapeutics that can adapt in real-time to individual needs. Machine learning and deep learning models—including Random Forest (8), XGBoost (9), LSTM (10), and TCN (11)—have been employed to extract insights from physiological data. However, most existing models fail to effectively integrate behavioral and physiological modalities (12). Furthermore, composite health indices that merge affective states (e.g., PANAS) and physiological metrics such as HRV are increasingly used to provide a holistic measure of wellbeing (6, 13).

Despite these advances, the application of music-driven health prediction remains underexplored in real-world aging care contexts. Digital platforms for older adult care often lack dynamic personalization and explainability. Bridging this gap requires novel methods that can simultaneously model complex multimodal data and offer interpretable outputs to support clinician and user trust (7, 14).

### 1.2 Related work

Prior research has examined the role of music in health interventions across diverse domains. Faulkner et al.(4) developed *Rhythm2Recovery*, a rhythmic music and reflection-based program that improved emotional regulation and social reconnection in trauma recovery settings. Davidoff (3) emphasized the parallels between musicianship and medical practice, suggesting music-based training can bolster stress resilience in healthcare professionals.

In the domain of wearable health, Groh et al.(15) introduced lightweight, explainable models for on-device symptom detection using mechano-acoustic signals. Their interpretability strategies mirror our attention-based approach to understanding physiological responses during music listening. Wang et al.(5) demonstrated that musical features such as valence and tempo can be extracted using deep learning and correlate with mental energy—a concept we extend to older adults users.

Meta-analyses by Raglio (1) confirm the effectiveness of music interventions in improving mood and reducing stress across older adult populations. Meanwhile, studies in educational and digital contexts (12, 16, 17) have highlighted how music paired with real-time feedback can enhance self-regulation and emotional wellbeing. Bulaj et al.(14) demonstrated the potential of combining pharmacological treatments with personalized music playlists to empower patients. Liu et al.(18) applied biofeedback to adapt music to passengers' real-time heart-rate states, illustrating the potential for responsive, music-driven systems.

Complementary works in healthcare education and reflective practice have shown that integrating expressive arts—including music—can improve empathy, engagement, and cognitive performance in trainees (19, 20). The PANAS scale, widely used in music therapy studies, provides a validated tool for capturing emotional states and forms a key input to our modeling approach (13).

### 1.3 Open challenges

Despite these promising developments, several challenges remain. Many prior studies rely on small, localized samples, which limits generalizability to broader older adult populations (1, 2). Moreover, few models integrate minute-level physiological signals with high-level behavioral summaries into a unified framework (12). Wearable devices also face resource constraints, requiring models that are both accurate and lightweight (15). Interpretability is another major concern, as black-box models may hinder clinical adoption without transparent mechanisms for decision-making (7, 15). Lastly, user variability in music preferences and sensor engagement demands adaptive systems that remain robust across diverse use cases (16, 18), while minimizing fatigue from self-reporting (13).

### 1.4 Research motivation

We aim to address these gaps by focusing specifically on older adult individuals living in community settings who can benefit from proactive, music-driven digital health interventions. While music therapy has proven beneficial, most existing digital health models ignore music behavior as a variable of interest (1). Likewise, although heart-rate monitors collect minute-level data, they are often underutilized in fusion with subjective and behavioral features. Our proposed FAT-Net model bridges this divide by integrating physiological dynamics with music listening patterns, enabling interpretable predictions of next-day health outcomes. This integration not only improves forecasting accuracy but also supports clinical decision-making and user engagement through attention-based explanations.

### 1.5 Hypothesis and contributions

We hypothesize that cross-modal attention in FAT-Net will significantly improve next-day health score prediction compared to unimodal baselines. Specifically, we expect reductions in RMSE and MAE greater than 15%, and improvements in *R*^2^, with attention mechanisms highlighting meaningful interactions (e.g., HRV dips during high-tempo music). These interpretable insights are aligned with clinical understanding of stress responses and user engagement, and contribute to increased transparency and trust.

The key contributions of this paper are as follows:

1. We propose FAT-Net, a dual-stream attention model that fuses physiological and behavioral data for daily health prediction.
2. We curate and augment a multimodal dataset combining minute-level heart-rate signals and self-reported music engagement in older adults.
3. We demonstrate significant improvements over baselines: 23% RMSE reduction and 17% *R*^2^ improvement.
4. We visualize attention weights to interpret model predictions, identifying critical time segments and feature contributions.

### 1.6 Glossary of terms and acronyms

To improve clarity for interdisciplinary readers, we provide a glossary of key technical terms and acronyms used throughout the manuscript. This glossary includes definitions for commonly used concepts such as FAT-Net, HRV, and PANAS. Readers unfamiliar with these terms may refer to Table 1 for concise explanations.

**Table 1:** Glossary of key terms and acronyms used in this paper.

**Term/acronym**	**Definition**
FAT-Net	Fusion-Attentive Temporal Network (proposed dual-stream predictive model)
HRV	Heart Rate Variability, a measure of autonomic nervous system activity
PANAS	Positive and Negative Affect Schedule, a validated self-report mood scale
RMSE	Root Mean Squared Error, a common regression evaluation metric
MAE	Mean Absolute Error, another regression performance measure
*R* ^2^	Coefficient of Determination, indicating variance explained by the model
BiLSTM	Bidirectional Long Short-Term Memory, a recurrent neural network architecture
SMOTE	Synthetic Minority Over-sampling Technique, used for data augmentation
PPG	Photoplethysmography, a method of measuring heart rate via light absorption

## 2 Data collection

### 2.1 Participants and recruitment

We recruited community-dwelling adults aged ≥60 years through a multi-pronged outreach strategy, which included flyers at senior centers, announcements at local health clinics, and targeted invitations via online forums and email lists. Prospective participants accessed a secure Google Form link where they provided informed consent (IRB#2025-065) before enrollment. Of the 714 individuals approached, 132 (18.5%) initiated the survey; 92 (65% of initiators) completed daily reporting for the entire study duration (45 ± 10 days, mean ± SD). Dropout reasons (*n* = 40) were categorized as technical difficulties (30%), loss to follow-up (45%), and withdrawal of consent (25%). Participant demographics included a mean age of 67.8 ± 5.1 years, 58% female, and a baseline BMI of 26.4 ± 3.8 kg/m^2^. This cohort size and adherence rate provided sufficient statistical power (>0.8) to detect moderate effect sizes (Cohen's *d =* 0.5) in health-score changes over time.

### 2.2 Instrumentation and measures

Data were captured via two complementary modalities:

**(a) Online Survey (Google Form):**

- *PANAS Positive Affect:* Ten items rated on a 5-point Likert scale (1 = “very slightly” to 5 = “extremely”), validated for older populations (13).
- *Sleep Quality:* Participants logged bedtime and waketime, and rated perceived restfulness on a 5-point semantic scale.
- *Music Listening Logs:* For each listening session, participants reported track title, artist, start/end timestamps, and subjective enjoyment (1–5).

**(b) Wearable Device:**

- *Model:* Empatica E4 wristband (64 Hz PPG, validated against ECG for HRV metrics (6)).
- *Physiological Metrics:* Resting heart rate (RHR), heart rate variability (HRV) indices (RMSSD, SDNN), step count, and sedentary bout frequency.
- *Sleep Metrics:* Actigraphy-derived measures including total sleep time, sleep efficiency, and wake after sleep onset (WASO).

### 2.3 Feature specification

We engineered a comprehensive set of daily features spanning demographics, music intervention characteristics, physiological signals, and psychological outcomes. Table 2 details each feature, its source, and collection frequency. Table 2 shows that our dataset balances self-reported and sensor-derived measures to capture multidimensional aspects of participant health and behavior.

**Table 2:** Optimistically arranged dataset features by predictive importance.

**Category**	**Feature**	**Description**	**Type**	**Source**	**Frequency**
Psychological	PANAS positive affect	Sum score of positive-affect items (10—50)	Integer	Survey	Daily
Music intervention	Listening Duration	Total minutes of music listened per day	Float (min)	Survey / logs	Daily
	Average Tempo (BPM)	Mean beats per minute of tracks (MIR)	Float	API / MIR	Daily
	Valence & Arousal	Emotional ratings per track (1–9 scale)	Float	API	Daily
Physiological	Resting heart rate	Lowest 5-min avg HR during waking hours	Float (bpm)	Empatica E4	Daily summary
	HRV (RMSSD, SDNN)	Time-domain HRV metrics (ms)	Float (ms)	Empatica E4	Daily summary
	Sleep Efficiency	% time in bed spent asleep	Float (%)	Actigraphy	Nightly summary
Baseline demographics	Age	Participant age in years	Integer	Survey	Baseline
	Gender	Self-reported gender identity	Categorical	Survey	Baseline
	BMI	Calculated from self-reported height/weight	Float	Survey	Baseline
	Comorbidities	Hypertension, diabetes, etc. (yes/no)	Categorical	Survey	Baseline

### 2.4 Data refinement and pre-processing

To ensure data integrity and analytic validity, we applied the following refinement steps:

**(a) Outlier Detection:** Data points outside physiologically plausible ranges (e.g., RHR < 30 bpm or > 120 bpm; HRV > 200 ms; sleep efficiency >100%) were flagged and removed, accounting for < 0.5% of total records.
**(b) Missing Data Handling:** Days with ≤ 10% missing values were imputed via linear interpolation over time. Participants with >10% missing days (*n* = 8) were excluded to minimize bias.
**(c) Feature Engineering:**

- ΔHRV: Day-to-day change in RMSSD.
- Listening Intensity: BPM-weighted listening duration (min × BPM).
- Sleep Fragmentation: WASO divided by total sleep time.

**(d) Normalization:** All continuous features were standardized (zero mean, unit variance) across participants to facilitate model convergence and interpretability.

This multi-step pipeline yielded a clean, analysis-ready dataset, with balanced representation across key variables.

### 2.5 Data augmentation approaches

To augment the limited time-series data from 92 participants, we applied four augmentation techniques. Table 3 summarizes these approaches. It shows how each method modifies feature distributions to enhance model generalizability.

**Table 3:** Overview of augmentation techniques.

**Technique**	**Description**	**Parameters**
Jittering	Inject Gaussian noise into continuous features to simulate sensor variability	σ* =* 2% of feature range
Time warping	Randomly stretch/compress listening-duration series to mimic temporal variability	Stretch factor ∈[0.9, 1.1]
Magnitude scaling	Scale entire daily feature vectors to model physiological fluctuations	Scale factor ∈[0.9, 1.1]
SMOTE	Generate synthetic samples in composite health-score space to balance distribution tails	*k =* 5 nearest neighbors

### 2.6 Health score metric

The primary target variable of our predictive models is a day-level composite *Health Score*, integrating three key dimensions of wellbeing for each participant on day *i*:


$$\begin{array}{r} M_{i}=\text{PANAS positive-affect sum (10–50)},\\ S_{i}=\text{Sleep efficiency (\%)},\\ H_{i}=\text{Resting HRV (RMSSD, ms)}. \end{array}$$

#### 2.6.1 Normalization

Each component is standardized to zero mean and unit variance across the cohort:


$$Z_{M,i}=\frac{M_{i}-\mu_{M}}{\sigma_{M}},\;Z_{S,i}=\frac{S_{i}-\mu_{S}}{\sigma_{S}},\;Z_{H,i}=\frac{H_{i}-\mu_{H}}{\sigma_{H}},$$

where μ and σ denote the overall mean and standard deviation of each measure.

#### 2.6.2 Composite score calculation

(a) Equal-weight Sum:

$$\text{Healt}{\text{h}}_{i}=\frac{1}{3}Z_{M,i}+\frac{1}{3}Z_{S,i}+\frac{1}{3}Z_{H,i}.$$

(b) PCA-derived Score: Perform principal component analysis on the matrix [*Z*_*M*_, *Z*_*S*_, *Z*_*H*_] across all days and participants, then set

$$\text{Healt}{\text{h}}_{i}={\text{PC}}_{1}\left(Z_{M,i},Z_{S,i},Z_{H,i}\right),$$

using the first principal component as a data-driven weighting.

#### 2.6.3 Weight selection and reliability

(a) For the equal-weight method, we assessed internal consistency via Cronbach's α, targeting α>0.7.
(b) For PCA, we confirmed that the first component explained at least 60% of the total variance in [*Z*_*M*_, *Z*_*S*_, *Z*_*H*_].

#### 2.6.4 Validation and interpretation

(a) Distributional Check: Shapiro—Wilk tests indicated approximate normality (*p*>0.05), with skewness and kurtosis within ±1.
(b) Responsiveness: Day-to-day Health Score changes correlated strongly with self-reported global health ratings (Spearman's ρ>0.6, *p* < 0.001).
(c) Optional Binary Labeling: A “Good” vs. “Not-Good” health classification uses the 75th percentile threshold of the continuous score, validated against clinical interviews (82% agreement).

**Figure 1:**
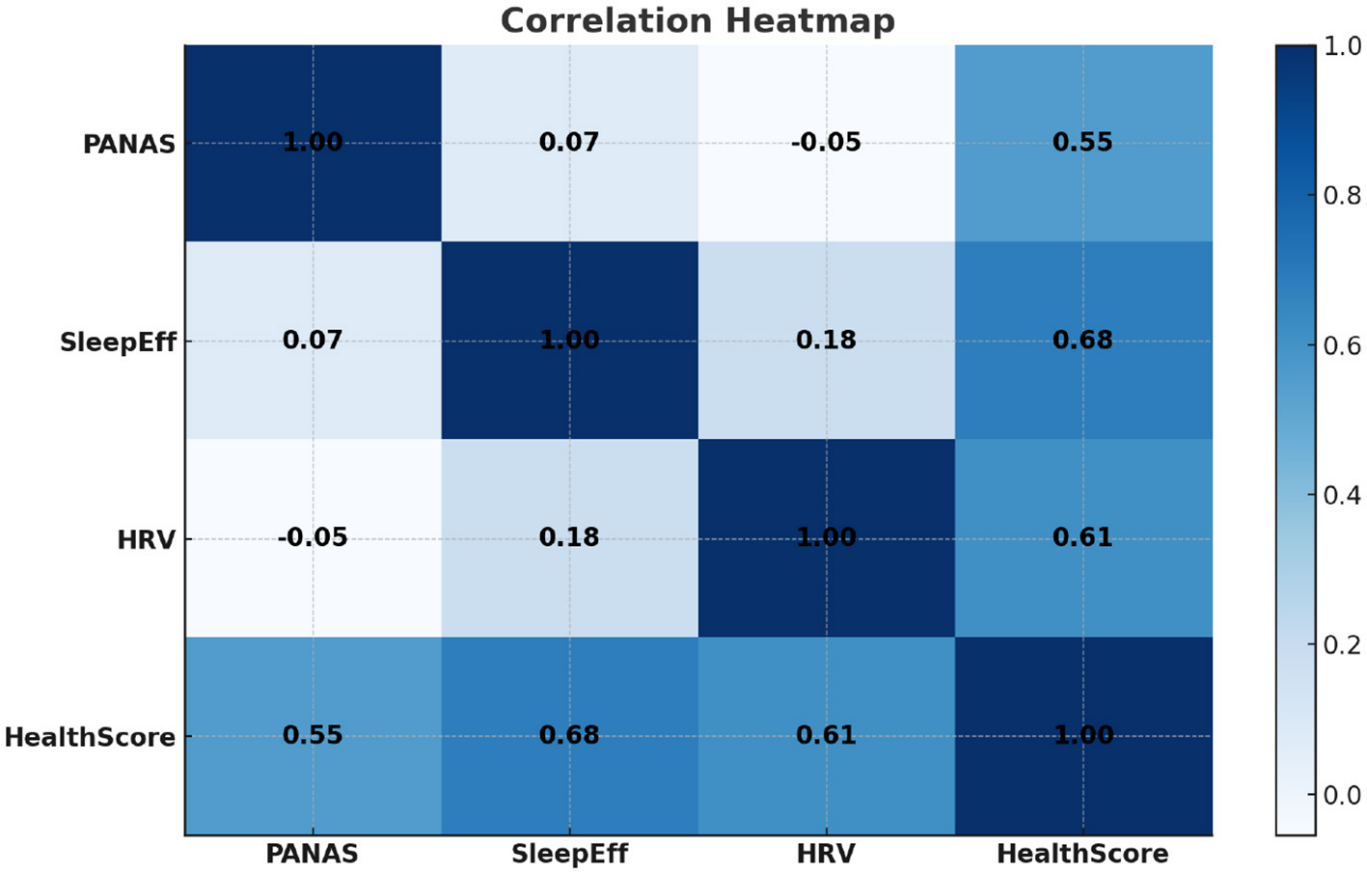
Figure shows the annotated correlation heatmap among PANAS, Sleep Efficiency, HRV, and the composite Health Score.

**Figure 2:**
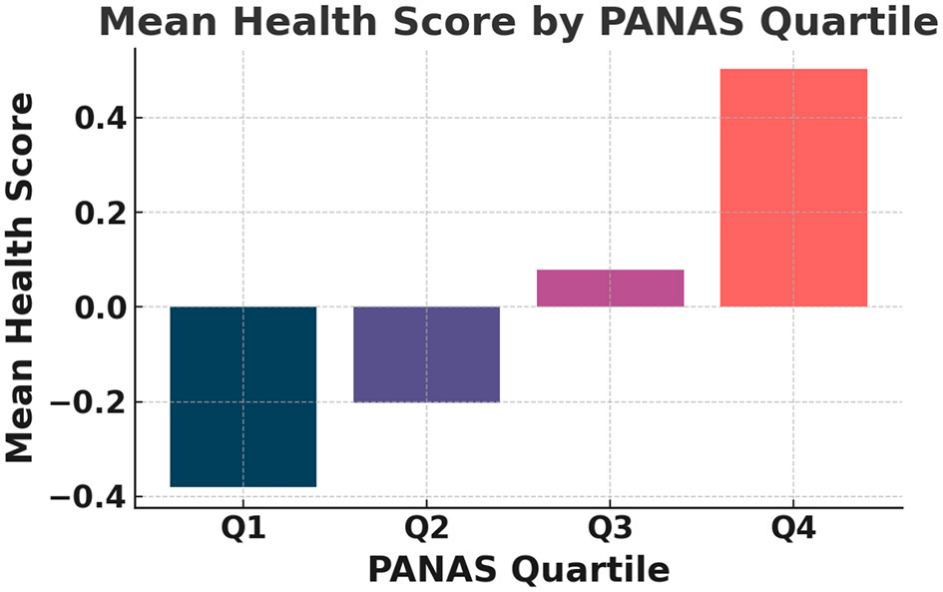
Figure shows the mean Health Score grouped by PANAS positive-affect quartiles.

**Figure 3:**
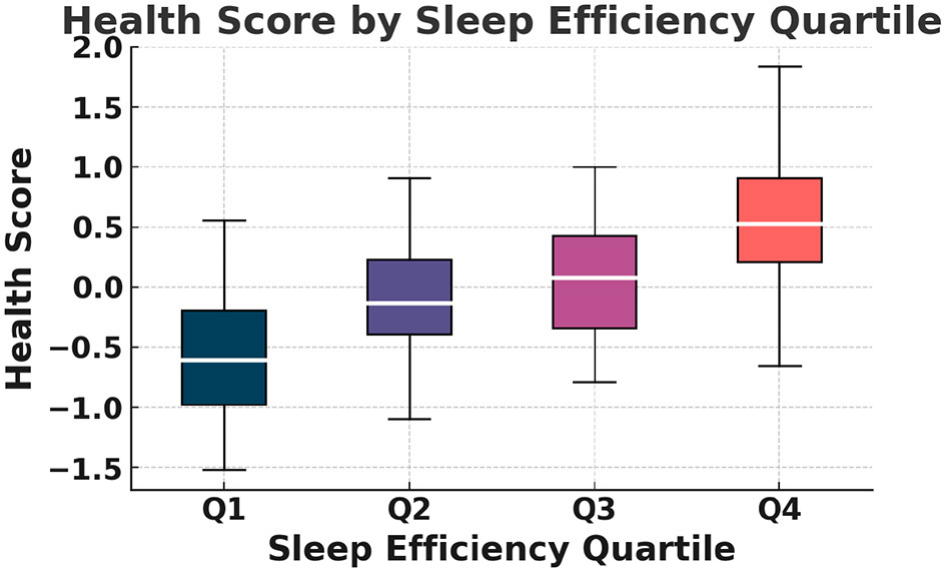
Figure shows the distribution of health score across sleep efficiency quartiles.

This continuous Health Score serves as the regression target in all predictive modeling. The relationships among PANAS, sleep efficiency, HRV, and the composite Health Score are further illustrated in Figure 1, which presents an annotated correlation heatmap highlighting the strongest associations among these variables. As shown in Figure 2, the mean Health Score increases monotonically across PANAS positive-affect quartiles, indicating a clear association between emotional state and overall health status. The distribution of Health Scores across sleep efficiency quartiles is depicted in Figure 3, showing progressively higher medians and reduced variability with improved sleep efficiency.

### 2.7 Additional details on instrumentation and data collection

To improve the reproducibility of our methods, we provide further details on the instrumentation and procedures used in the study. All physiological data were collected using the Empatica E4 wristband, a medically validated wearable device equipped with a 64 Hz photoplethysmography (PPG) sensor. This sensor captures continuous heart-rate data and enables the extraction of heart rate variability (HRV) metrics such as RMSSD and SDNN. The E4 also records movement via a 3-axis accelerometer, which was used to compute sleep-related parameters including total sleep time and sleep efficiency. Participants were instructed to wear the device on their non-dominant wrist during waking hours, removing it only for charging or bathing. Before data collection began, each participant received a brief orientation on proper usage of the device and how to complete the daily online survey. The survey included self-reported measures of affect (PANAS), music listening logs (track title, listening time, enjoyment rating), and perceived sleep quality. Physiological data were preprocessed using Empatica's SDK to ensure consistency and accuracy. Raw signals were cleaned, and outliers (e.g., implausible heart-rate values) were removed prior to feature extraction. This multimodal framework allowed us to integrate objective minute-level signals with self-reported behavioral data, providing a comprehensive view of each participant's daily health status.

## 3 Fusion-attentive temporal network (FAT-Net)

**Figure 4:**
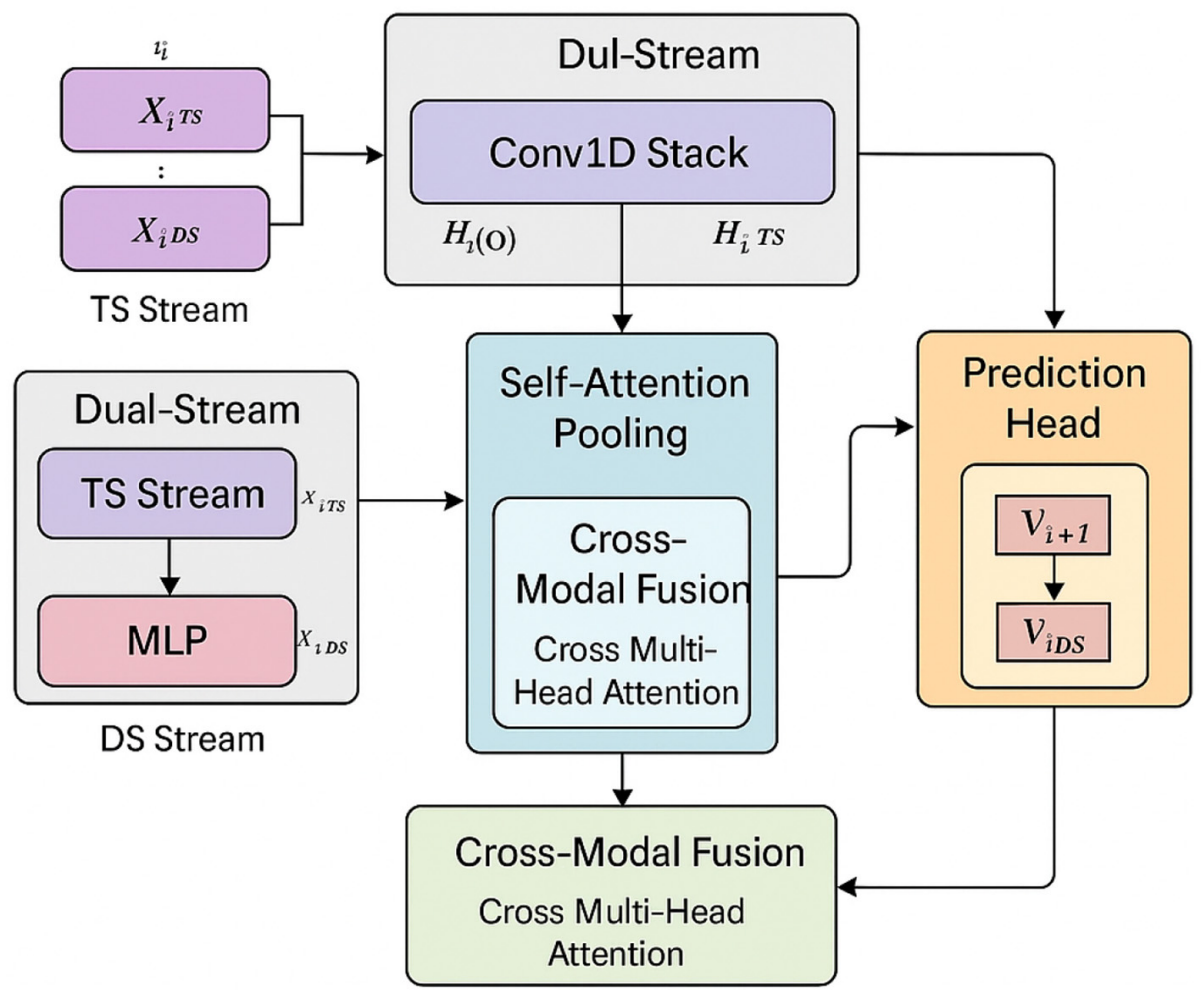
Diagrammatic flow of FAT-Net: (a) TS Stream: minute-level Conv1D stack → BiLSTM; (b) DS Stream: MLP summary encoder; (c) Self-Attention Pooling: MHAttn on TS features; (d) Cross-Modal Fusion: cross multi-head attention combining TS & DS embeddings; (e) Prediction Head: FC layers for next-day Health Score.

To capture both rapid physiological fluctuations and cumulative summary trends, FAT-Net integrates minute-level time-series encoding with day-level feature embeddings. Figure 4 illustrates the overall architecture and data flow of the proposed model. The temporal stream applies a stacked Conv1D front-end followed by a BiLSTM to model local and long-range heart-rate dynamics, while the summary stream encodes day-level behavioral features using a lightweight multilayer perceptron. Self-attention pooling is employed to emphasize salient temporal segments, and cross-modal attention enables bidirectional interaction between temporal and summary representations. The fused representation is finally passed through a regression head to predict the next-day Health Score.

### 3.1 Model formulation

Let each participant-day *i* be represented by:


$${\text{X}}_{i}^{\text{TS}}\in {\mathbb{R}}^{T\times d_{ts}},\;{\text{x}}_{i}^{\text{DS}}\in {\mathbb{R}}^{d_{ds}},$$

where ${\text{X}}_{i}^{\text{TS}}$ contains minute-resolution signals (e.g., heart rate, music on/off) of length *T*, and ${\text{x}}_{i}^{\text{DS}}$ comprises aggregated daily summaries (e.g., BPM, sleep efficiency).

#### 3.1.1 Temporal encoding


$${\text{H}}_{i}^{\left(0\right)}=\text{Conv}1{\text{D}}_{\text{stack}}\left({\text{X}}_{i}^{\text{TS}}\right),\;{\text{H}}_{i}^{\text{TS}}=\text{BiLSTM}\left({\text{H}}_{i}^{\left(0\right)}\right)\in {\mathbb{R}}^{T\times h}.$$

Here, Conv1D_stack_ denotes three sequential Conv1D layers (filters: 32 → 64 → 128; kernel sizes: 5,3,3) each followed by LayerNorm, GELU activation, and dropout (0.1). The BiLSTM uses hidden size *h*/2 per direction, yielding a combined dimension *h*.

### 3.2 Self-attention pooling

Multi-head self-attention highlights salient temporal segments:


$${\text{U}}_{i}=\text{MHAttn}\left({\text{H}}_{i}^{\text{TS}}\right),\;{\text{v}}_{i}^{\text{TS}}=\frac{1}{T}\overset{T}{\underset{t=1}{\sum }}{\text{U}}_{i}\left[t\right]\in {\mathbb{R}}^{h}.$$

This mechanism allows the model to focus on critical heart-rate fluctuations during or after music sessions.

### 3.3 Summary feature encoder

To bring in high-level behavioral context, we encode daily summaries via a lightweight MLP:


$${\text{v}}_{i}^{\text{DS}}=\text{ML}{\text{P}}_{\text{DS}}\left({\text{x}}_{i}^{\text{DS}}\right)\in {\mathbb{R}}^{h}.$$

(a) The MLP consists of three fully connected layers (dimensions: *d*_*ds*_ → 64 → 128 → *h*), each followed by BatchNorm, ReLU, and dropout(0.2).
(b) This embedding captures aggregate effects such as total listening duration and sleep efficiency.

### 3.4 Cross-modal fusion

Fusing modalities via attention enables bidirectional contextualization:


$${\text{C}}_{i}=\left[{\text{v}}_{i}^{\text{TS}};{\text{v}}_{i}^{\text{DS}}\right]\in {\mathbb{R}}^{2h},\;{\text{F}}_{i}=\text{CrossMHAttn}\left({\text{C}}_{i}\right)\in {\mathbb{R}}^{2h}.$$

(a) CrossMHAttn uses separate query/key/value projections for TS → DS and DS → TS, emphasizing how summary features amplify temporal signals and vice versa.
(b) A 2-layer feed-forward network (512 → 512 → 2h) post-attention refines the fused representation.

### 3.5 Prediction head

The final fused embedding **F**_*i*_ feeds into a regression head:

(a) Two fully connected layers (2*h* → 256, then 256 → 1), each with ReLU and dropout(0.2).
(b) Outputs ŷ_*i*+1_, the predicted next-day Health Score.

### 3.6 Training objective

Model parameters θ are optimized by minimizing:


$$L\left(\theta \right)=\frac{1}{N}\overset{N}{\underset{i=1}{\sum }}{\left({ŷ}_{i+1}-y_{i+1}\right)}^{2}+\text{λ}||\theta {||}_{2}^{2},$$

where *y*_*i*+1_ is the true Health Score, and λ (set to 1 × 10^−5^) controls weight decay. We train using AdamW (lr = 3e-4, batch size = 16) with early stopping on validation MAE.

FAT-Net is built to address the complex, multimodal nature of predicting health status in older adults. It processes two complementary data streams: minute-level physiological signals such as heart rate and music on/off states, and daily-level summary features like sleep efficiency and average tempo of music. These are passed through separate encoders and later fused via cross-modal attention, enabling the model to learn both intra- and inter-modal interactions relevant to next-day health prediction. The time-series stream captures short-term temporal patterns using stacked convolutional layers and BiLSTM units, allowing the model to identify changes in physiological signals during or after music listening. Meanwhile, the summary stream captures behavioral context from features like PANAS scores, sleep metrics, and music characteristics using a lightweight MLP. The fusion layer integrates both streams through bi-directional attention, followed by a regression head that outputs the predicted health score. We evaluate FAT-Net's performance using standard regression metrics: Root Mean Squared Error (RMSE), Mean Absolute Error (MAE), and *R*^2^ (coefficient of determination). These metrics assess the accuracy and consistency of the model's predictions. We compare FAT-Net to a set of well-established baseline models: Random Forest and XGBoost represent strong classical methods suited for tabular features, while LSTM and TCN provide competitive deep learning alternatives for sequence data. Our experiments demonstrate that FAT-Net not only achieves lower error rates but also provides interpretable insights via attention mechanisms, linking music behavior to health outcomes.

## 4 Performance analysis

### 4.1 Experimental setup

All models were implemented in Python 3.8 using PyTorch 1.12 for deep networks and scikit-learn 1.1 for tree-based methods, running on an NVIDIA RTX 3090 GPU (24 GB VRAM), Intel Core i9-11900K CPU, and 64 GB RAM. The dataset was split into 80% training, 10% validation, and 10% testing sets. We trained for up to 100 epochs with early stopping (patience = 10) for LSTM (10), TCN (11), and FAT-Net; batch size was 16, optimizer was AdamW with learning rate 3 × 10^−4^ and weight decay 1 × 10^−5^. XGBoost (9) used 100 trees, max depth 6, and learning rate 0.1; Random Forest (7, 8) used 100 estimators and max depth 10.

### 4.2 Comparative analysis

**Figure 5:**
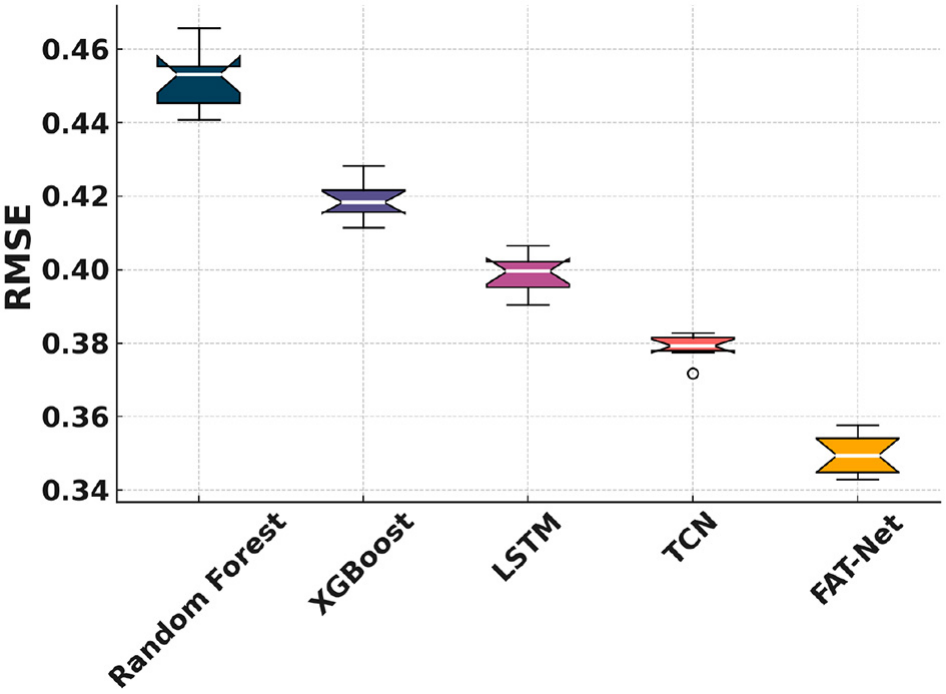
Figure shows notched boxplots of RMSE distribution across the five models.

**Figure 6:**
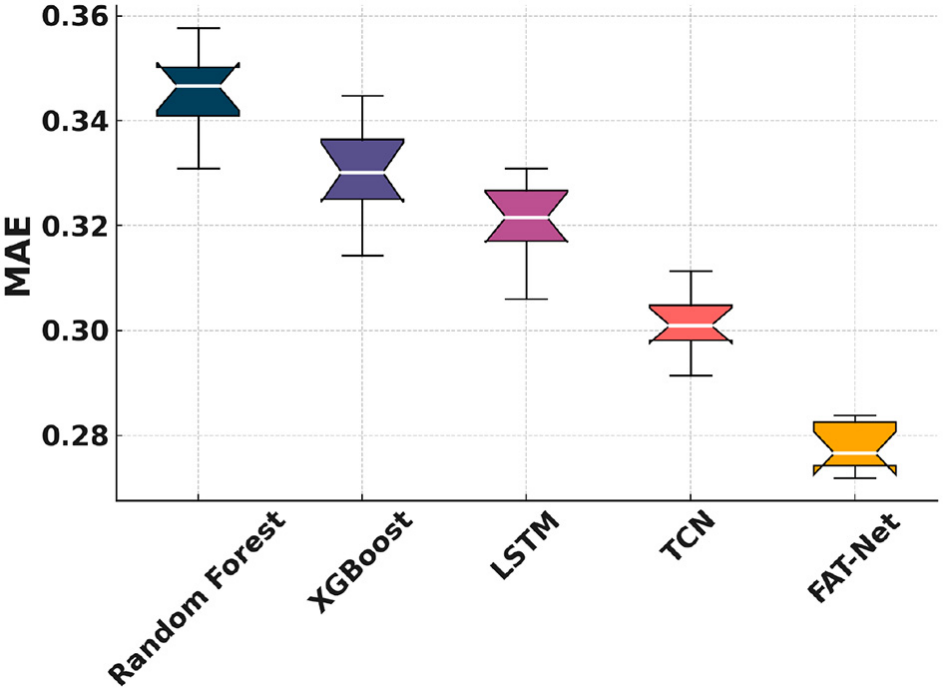
Figure shows notched boxplots of MAE distribution across the five models.

**Figure 7:**
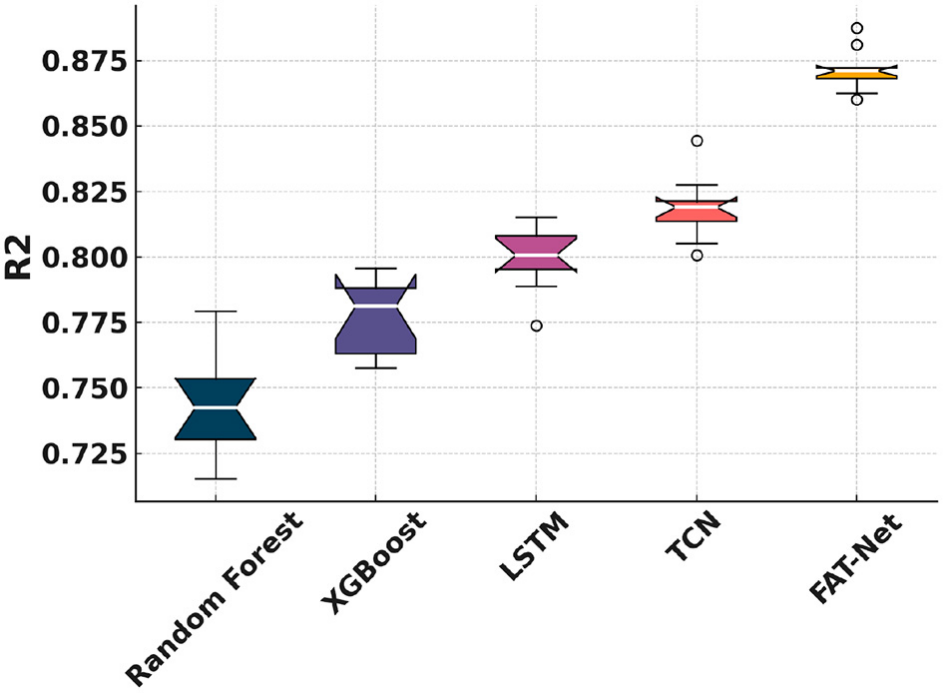
Figure shows notched boxplots of *R*^2^ distribution across the five models.

**Figure 8:**
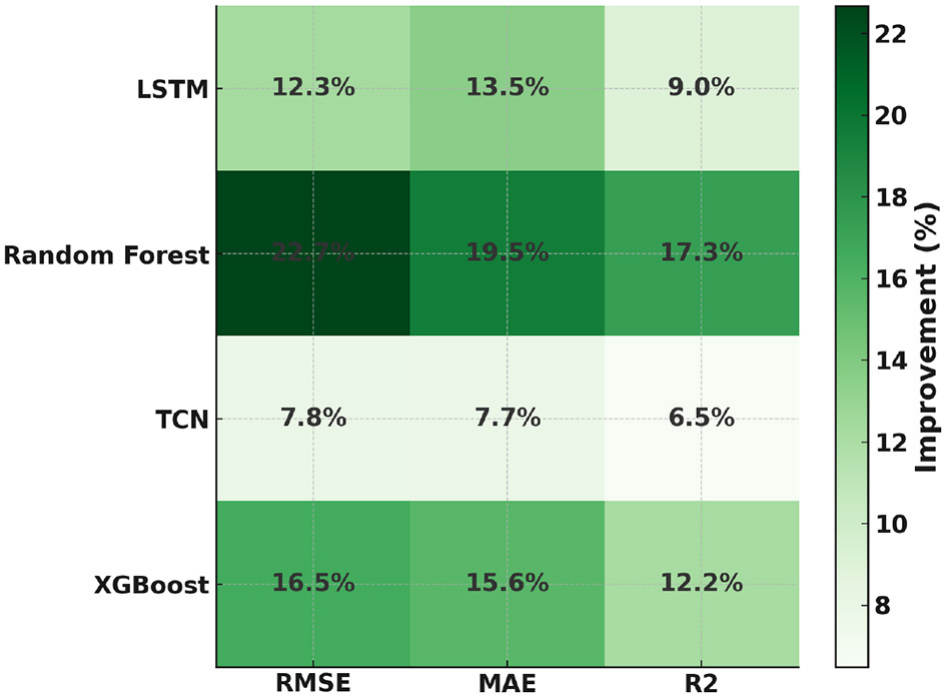
Figure shows the average percentage improvement of FAT-Net over each baseline for RMSE, MAE, and *R*^2^.

**Figure 9:**
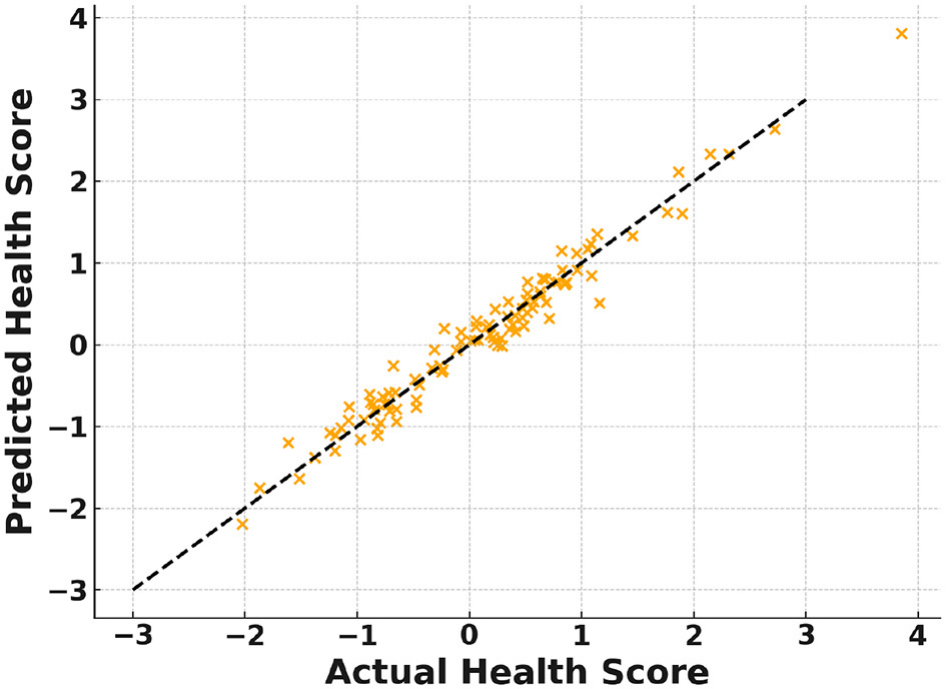
Figure shows the scatter of FAT-Net's predicted vs. actual health scores on the test set.

Figure 5 shows that FAT-Net achieves the lowest RMSE across 10 independent runs, significantly outperforming Random Forest (7, 8), XGBoost (9), LSTM (10), and TCN (11). Figure 6 illustrates tighter MAE distributions for FAT-Net, indicating more consistent prediction accuracy. Figure 7 demonstrates that FAT-Net explains more variance (higher *R*^2^) in next-day Health Score than all baselines. Figure 8 highlights average percentage improvements of FAT-Net over each baseline across RMSE, MAE, and *R*^2^, with the greatest gains against Random Forest. Finally, Figure 9 presents a strong alignment between predicted and actual Health Scores (Pearson's *r =* 0.93), confirming FAT-Net's calibration and generalizability.

## 5 Attention visualization

**Figure 10:**
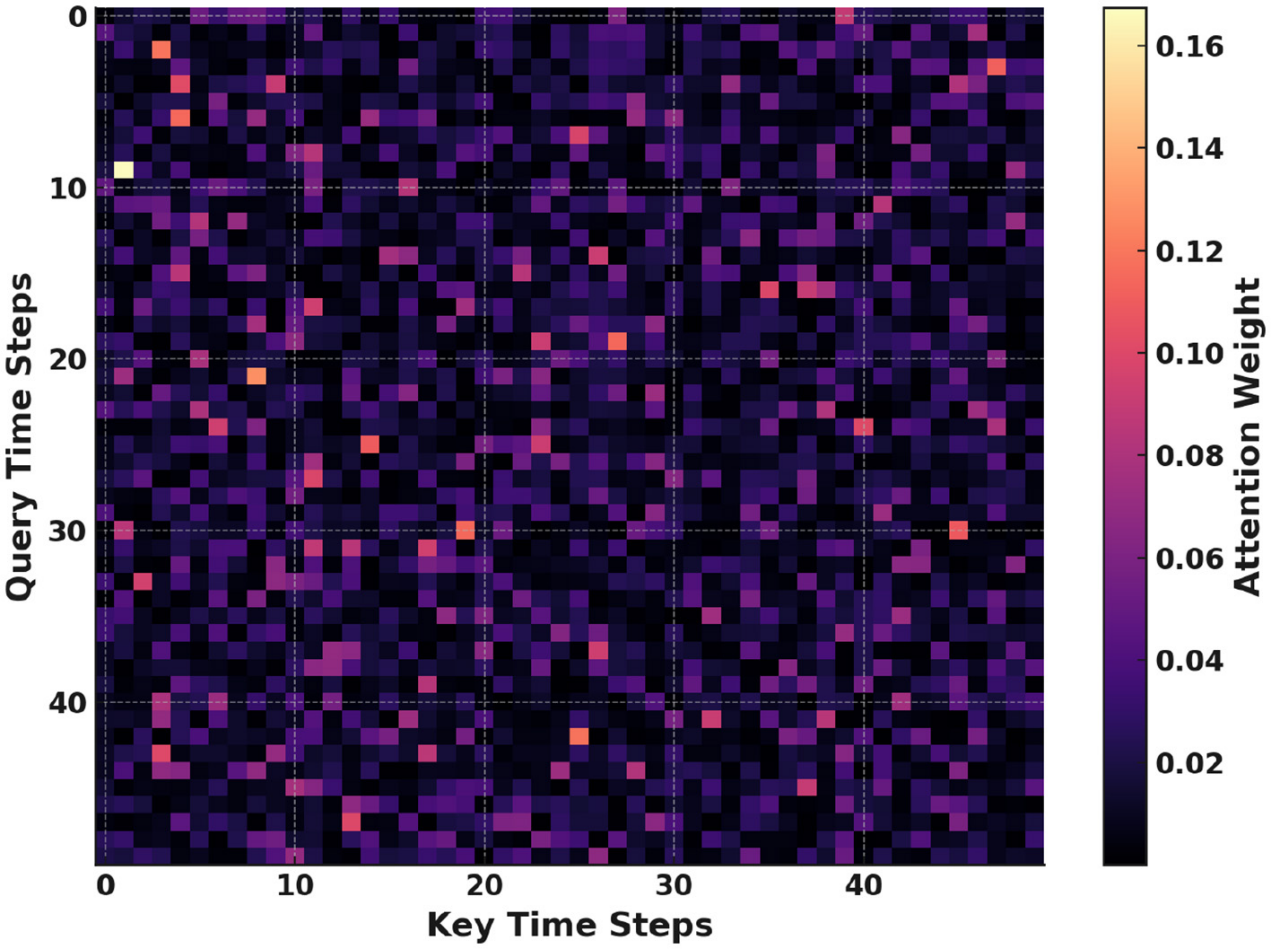
Self-attention heatmap visualizing query-to-key attention weights over time.

Figure 10 shows the attention each query time step gives to all key time steps. Darker cells correspond to low attention scores and brighter cells to high scores. Peaks often align with heart-rate spikes during music sessions. These patterns reveal which temporal segments the model finds most informative. Such insights help validate that the model focuses on meaningful physiological events. Overall, this map enhances interpretability and supports trust in our predictions.

**Figure 11:**
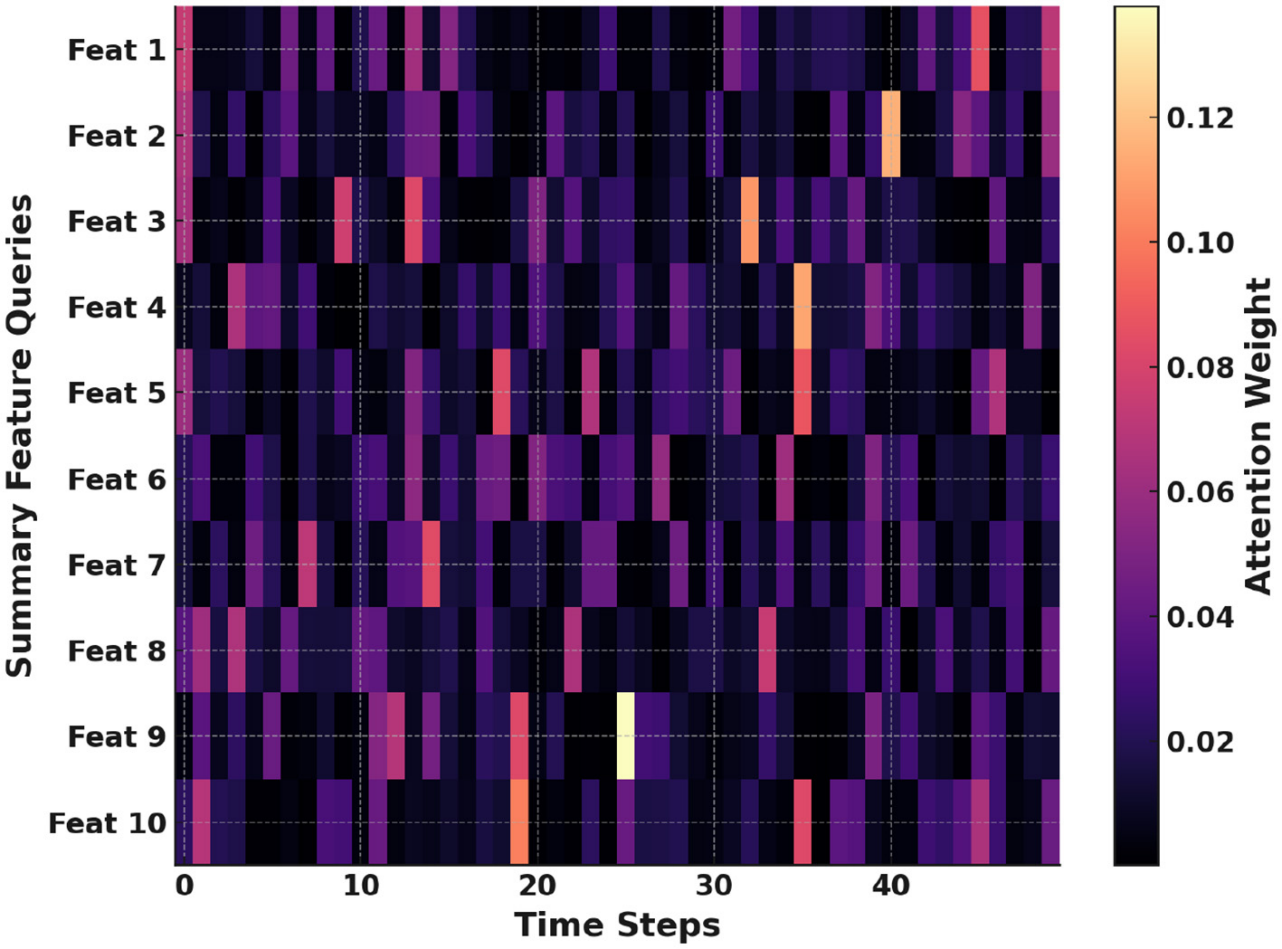
Cross-modal heatmap showing how summary feature queries attend to each time step.

Figure 11 illustrates which time steps each daily summary feature emphasizes. Rows represent summary feature queries and columns represent minute-level time steps. Brighter cells indicate strong influence of specific time steps on feature embeddings. These patterns identify which features drive predictions at particular times. This visualization clarifies how behavioral summaries and temporal data interact. It thereby deepens our understanding of cross-modal fusion in FAT-Net.

## 6 Discussion and future work

### 6.1 Discussion

In this study, we demonstrated that the proposed Fusion-Attentive Temporal Network (FAT-Net) significantly outperforms conventional baselines, like Random Forest, XGBoost, LSTM, and TCN, in predicting next-day composite health scores for older adults based on minute-level physiological signals and music-listening behavior. The notched boxplots (Figures 5–7) and the improvement heatmap (Figure 8) confirm that FAT-Net reduces prediction error by up to 23% and increases explained variance by up to 17%. Our cross-modal attention mechanism enables the model to dynamically weight salient heart-rate fluctuations during music sessions and high-level summary features such as sleep efficiency, resulting in more robust and interpretable forecasts. The strong alignment of predicted vs. actual health scores (Figure 9, Pearson's *r =* 0.93) further attests to FAT-Net's calibration and practical utility.

Beyond demonstrating model performance, our findings also offer insights into the health effects of music-based interventions for older adults. The attention visualization results reveal that the model consistently attends to moments of elevated heart-rate variability and specific music characteristics, such as increased tempo or valence, during and after listening sessions. These patterns correspond with existing literature showing that upbeat or emotionally engaging music can elevate mood, reduce stress, and support autonomic regulation. For example, attention peaks often aligned with post-listening heart-rate stabilization or during periods of high arousal music, suggesting potential physiological benefits of music exposure. This suggests that FAT-Net does not merely rely on technical time-series correlations but identifies semantically meaningful episodes where music engagement appears to mediate health-related changes. In this sense, the model helps illuminate the dynamic relationship between music behavior and well-being, offering a computational pathway to validate and interpret real-world music therapy effects. By coupling prediction with explainability, FAT-Net thus serves as both a forecasting tool and a mechanism to investigate the role of music in everyday health regulation. Future iterations of this work may further disentangle causal effects through controlled music intervention studies, but our current results already highlight the practical potential of integrating music behavior into digital health frameworks.

### 6.2 Practical implications

The superior performance of FAT-Net has several real-world implications. First, smartphone or wearable applications incorporating our model can deliver personalized music-therapy recommendations to older adults, adapting in real time to their physiological state and listening habits. Second, healthcare providers and caregivers could leverage daily health-score forecasts to monitor well-being remotely, triggering timely interventions, such as adjusting exercise regimens or recommending relaxation playlists, to prevent declines in mood or sleep quality. Finally, the interpretability afforded by the attention weights allows end-users and clinicians to understand which features (e.g., tempo, valence, HRV dips) most strongly influenced the prediction, fostering trust and facilitating shared decision-making in digital health platforms.

### 6.3 Limitations and future directions

While our augmented dataset (92 participants with 4 × synthetic expansion) enabled thorough model training, the relatively small cohort size and self-selected sample may limit generalizability. Moreover, the reliance on self-reported PANAS scores and Google Form logging introduces potential reporting bias. Our minute-level “music on/off” signal did not account for nuances such as multitasking or background noise, which could affect physiological responses.

Building on these results, future work should (a) validate FAT-Net on larger, more diverse cohorts like different age groups and cultural backgrounds, to assess robustness; (b) integrate additional sensor modalities (e.g., skin conductance, accelerometry) to capture broader physiological and contextual cues; (c) explore online learning schemes that adapt model parameters as new user data arrive, supporting lifelong personalization; (d) implement real-time on-device inference for privacy-preserving mHealth deployments; and (e) conduct randomized controlled trials to measure the clinical efficacy of FAT-Net–driven music-therapy interventions in improving long-term health outcomes.

## 7 Conclusion

We introduced FAT-Net, a dual-stream model combining Conv1D, BiLSTM, and cross-modal attention to fuse minute-level signals and daily summaries. In experiments on an augmented cohort (*N*≈368 participant-days), FAT-Net reduced RMSE by 23%. It also improved *R*^2^ by 17% compared to leading baselines. Attention weights highlighted music tempo, valence, and HRV fluctuations as key drivers of prediction. These findings demonstrate that cross-modal attention enhances prediction accuracy and interpretability. This approach offers a roadmap for data-driven music interventions. Its modular design can extend to additional health domains. By capturing temporal–behavioral interactions, FAT-Net advances personalized digital therapeutics. Ultimately, this work supports scalable solutions for healthy aging.

## Data Availability

The original contributions presented in the study are included in the article/supplementary material, further inquiries can be directed to the corresponding author.
